# Advances in CRISPR-Cas12a/13a-Based Nucleic Acid Detection for Porcine Viral Diseases: A Comprehensive Review

**DOI:** 10.3390/vetsci13020141

**Published:** 2026-01-31

**Authors:** Xianyu Zhang, Xin Zhao, Yating Song, Yuewen Luo, Li Yao, Qiaolin Wu, Tingzhang Ye, Wanqin Liang, Xiaoyu Zhang, Yingyu Liang, Baizheng Liang, Jingyan Zhang, Xiangyang Li

**Affiliations:** 1 School of Food & Pharmaceutical Engineering, Zhaoqing University, Zhaoqing 526061, China; 2010020066@zqu.edu.cn; 2 Zhaoqing Animal Disease Control Center, Zhaoqing 526000, China

**Keywords:** swine viral diseases, CRISPR-Cas12a, CRISPR-Cas13a, isothermal amplification, point-of-care diagnostics, nucleic acid detection

## Abstract

The global swine industry suffers persistent economic losses and health challenges due to major viral pathogens such as African swine fever virus (ASFV), porcine reproductive and respiratory syndrome virus (PRRSV), classical swine fever virus (CSFV), and porcine circovirus (PCV). Traditional diagnostic methods, including virus isolation, serology, and quantitative PCR (qPCR), are limited by time, equipment requirements, and field applicability. Recent advances in CRISPR-based diagnostics, particularly those leveraging the collateral cleavage activity of Cas12a and Cas13a, have enabled rapid, sensitive, and field-deployable nucleic acid detection. This review outlines the principles of CRISPR-Cas12a/13a systems, their integration with isothermal amplification techniques, and their application in detecting major swine viruses. Cas12a-based platforms (e.g., DETECTR) and Cas13a-based systems (e.g., SHERLOCK) achieve detection limits as low as single-copy/μL within 25–60 min at 37 °C, offering high specificity and compatibility with visual readouts. Applications include ASFV, PRRSV, CSFV, PCV, foot-and-mouth disease virus (FMDV), porcine rotavirus (PoRV), and porcine parvovirus 7 (PPV7). Despite significant advances, challenges remain, notably the reliance on nucleic acid extraction and the need for fully integrated “sample-in, result-out” systems. Ongoing innovations in extraction-free methods, lyophilized reagents, and multiplex detection will strengthen the role of CRISPR diagnostics in swine disease surveillance and control. From an application standpoint, the technology offers a low-capital, field-adaptable alternative to qPCR, with its value proposition rooted in early outbreak containment and loss prevention. Its adoption pathway is expected to vary across production systems—serving as a sentinel tool in intensive settings, a leapfrogging solution in rapidly intensifying regions, and through shared-service models in resource-limited contexts. However, translation to routine use still requires overcoming standardization hurdles, regulatory validation, and workflow integration.

## Simple Summary

Swine viral diseases cause severe economic losses to the global pig industry. Traditional diagnostic methods often require complex equipment and take a long time, which limits their use for rapid on-site testing. This review discusses a new detection technology based on CRISPR-Cas systems, gene-editing tools that act like molecular scissors to precisely recognize viral genetic material. When combined with constant-temperature amplification methods, this approach can detect important swine viruses—such as African swine fever virus and porcine reproductive and respiratory syndrome virus—with high sensitivity and specificity within one hour. The test does not need expensive instruments and can be read using a simple test strip or a portable fluorescent device. Although sample processing steps are still required, this technology offers a breakthrough solution for fast, on-site diagnosis. It can help control outbreaks more quickly, protect animal health, and safeguard pork supply chains, thereby supporting the stability and sustainability of pig farming worldwide.

## 1. Introduction

The global swine industry faces ongoing threats from viral pathogens that cause substantial economic losses through mortality, reduced productivity, trade restrictions, and control costs [1]. Among the most impactful are African swine fever virus (ASFV), porcine reproductive and respiratory syndrome virus (PRRSV), classical swine fever virus (CSFV), and porcine circovirus type 2 (PCV2). These viruses differ in transmission dynamics, clinical manifestations, and potential for rapid spread, highlighting the critical need for timely and accurate diagnostics to enable effective outbreak management and bio-security [2].

Conventional diagnostic approaches include virus isolation (which offers high specificity but is slow and technically demanding), serological assays such as ELISA (useful for surveillance but limited in early infection), and molecular methods like qPCR, which provides high sensitivity and specificity but requires sophisticated equipment and skilled personnel [3,4]. Isothermal amplification techniques, including loop-mediated isothermal amplification (LAMP) and recombinase polymerase amplification (RPA), have emerged as promising field-deployable alternatives. However, they are often constrained by non-specific amplification and the need for nucleic acid extraction [5].

The discovery of CRISPR-Cas systems has revolutionized molecular diagnostics. Cas12a and Cas13a, in particular, exhibit unique collateral cleavage activity upon recognition of specific DNA or RNA targets, enabling signal amplification through non-specific degradation of fluorescent reporter molecules [6,7,8]. When combined with isothermal pre-amplification, these systems achieve attomolar sensitivity, single-base specificity, and rapid readouts via fluorescence or lateral flow strips—all under constant temperature conditions suitable for point-of-care use. Platforms such as DETECTR (DNA Endonuclease Targeted CRISPR Trans Reporter) [6] and SHERLOCK (Specific High-sensitivity Enzymatic Reporter unLOCKing) [9] exemplify the potential of CRISPR-based diagnostics for infectious disease surveillance.

This review synthesizes recent advances in CRISPR-Cas12a/13a technologies, detailing their mechanisms, technical optimization, and applications in detecting major swine viruses. We highlight performance metrics, field adaptability, and remaining challenges, providing a comprehensive resource for researchers and veterinarians engaged in swine disease diagnostics and control.

## 2. Global Burden and Diagnostic Landscape of Major Porcine Viral Diseases

**Figure 1:**
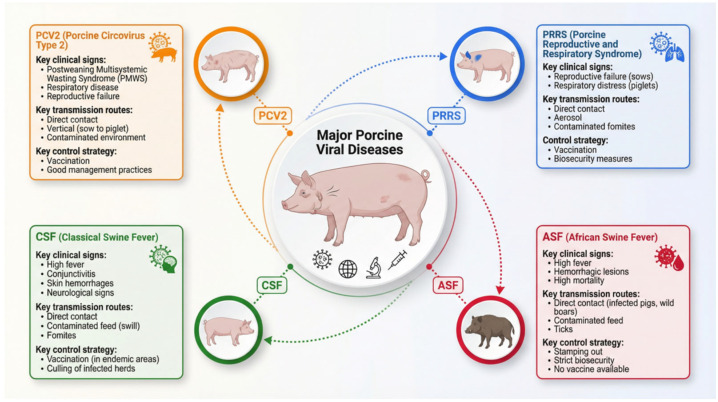
Key characteristics of economically significant porcine viral pathogens. Schematic comparison of four major swine viral diseases: Porcine Reproductive and Respiratory Syndrome (PRRS), African Swine Fever (ASF), Classical Swine Fever (CSF), and Porcine Circovirus Type 2 (PCV2). Summary of key clinical signs associated with each pathogen, highlighting distinct symptomatic profiles. Primary transmission routes for each virus, illustrating modes of spread within and between herds. Recommended control strategies, including bio-security measures, vaccination, and outbreak containment protocols.

The global swine industry continues to confront severe economic challenges due to viral pathogens that compromise animal health, productivity, and international trade [1]. Porcine Reproductive and Respiratory Syndrome (PRRS), African Swine Fever (ASF), Classical Swine Fever (CSF), and Porcine Circovirus Type 2 (PCV2)-associated diseases (PCVAD) are particularly consequential, each presenting unique virological complexities and significant disease management hurdles [10,11] (Figure 1). The recent emergence of novel strains, shifting epidemiological patterns, and recurrent outbreaks underscore the necessity for robust control strategies [2,12,13]. In an era of intensified global trade, the demand for effective, rapid, and accurate diagnostic tools has become increasingly urgent [14]. The economic impacts of these diseases are profound and multifaceted. Direct losses arise from high mortality, reduced growth performance, reproductive failures, and depopulation costs, while indirect losses result from trade restrictions, stringent bio-security measures, and investments in vaccination and surveillance programs [15,16,17]. For instance, the ASF pandemic since 2018 has led to catastrophic losses in Asia and Europe, necessitating the culling of millions of pigs and disrupting global pork markets [18,19]. PRRS remains endemic in major swine-producing regions, causing annual losses estimated in the hundreds of millions to billions of dollars. Similarly, CSF outbreaks can lead to high mortality and strict trade embargoes [20], while PCV2, though often controlled by vaccination, continues to cause substantial economic impact through sub-clinical infections and production losses [21].

Effective disease control relies on timely and precise diagnosis. The diagnostic arsenal for these viruses has evolved to include virological, serological, and molecular methods, each with distinct advantages and limitations. Virus Isolation (VI) remains a gold standard for confirmatory diagnosis, offering high specificity. It is formally recommended for ASF and CSF detection [22,23]. However, VI is technically demanding, time-consuming (often requiring days to weeks), and reliant on viable virus and susceptible cell cultures, making it unsuitable for high-throughput screening or rapid field deployment [23]. Serological assays, primarily Enzyme-Linked Immunosorbent Assays (ELISAs), are valuable for detecting host antibody responses, aiding in serosurveillance, proving freedom from infection, and monitoring vaccine efficacy. Antibody detection is a cornerstone for ASF, CSF, and PRRS surveillance [22,23,24]. A key limitation is the diagnostic window period; antibodies typically appear days to weeks post-infection, rendering these methods ineffective for detecting acute infections before seroconversion [23,25]. For PCV2, antibody detection methods such as ELISA are useful for monitoring herd exposure but cannot definitively diagnose active infection due to potential interference from vaccine-induced antibodies [2].

The advent of Polymerase Chain Reaction (PCR)-based technologies revolutionized porcine viral diagnostics. Conventional and, more importantly, real-time quantitative PCR (qPCR) have become frontline molecular tools due to their high sensitivity, specificity, rapid turnaround, and ability to quantify viral load. qPCR is extensively validated and recommended by the World Organisation for Animal Health (WOAH) for diseases such as ASF, CSF, and PRRS [10,22,23]. For PCV2 diagnosis, qPCR is important for detecting viral presence in tissues and fluids, helping to distinguish clinical from subclinical cases. Despite its strengths, qPCR’s dependence on sophisticated thermal cycling equipment, stable power, and trained personnel confines its use to well-equipped central laboratories, limiting its application in resource-limited settings or for point-of-need testing (PONT) [10].

To address the need for field-deployable tools, isothermal amplification techniques have been developed. Methods such as Loop-Mediated Isothermal Amplification (LAMP) and Recombinase Polymerase Amplification (RPA) amplify nucleic acids at a constant temperature, eliminating the need for thermal cyclers [10]. When coupled with simple visual readouts (e.g., color change or lateral flow strips), they offer significant potential for on-site use. Assays based on LAMP or RPA have been reported for ASFV, PRRSV, and other viruses [10]. Nevertheless, challenges persist, including the risk of non-specific amplification leading to false positives, the complexity of primer design for some methods, and the frequent requirement for nucleic acid extraction, which remains a bottleneck for true simplicity and speed [26].

In summary, the current diagnostic paradigm for major porcine viruses employs a tiered approach: serology for population-level surveillance, qPCR for sensitive and quantitative confirmation in laboratories, and isothermal methods as promising yet not fully optimized field alternatives. A notable gap exists for a diagnostic platform that integrates the high sensitivity and specificity of qPCR with the simplicity, speed, and equipment independence of ideal isothermal amplification, while providing a robust and user-friendly visual readout. This unmet need for accurate, rapid, portable, and cost-effective diagnostics represents a niche where novel CRISPR/Cas-based detection systems, particularly those leveraging the trans-cleavage activity of Cas12a, are emerging as transformative technologies, promising to bridge the gap between centralized laboratory testing and field-based decision-making.

## 3. Principles and Technical Features of CRISPR/Cas12a-Based Detection

**Figure 2:**
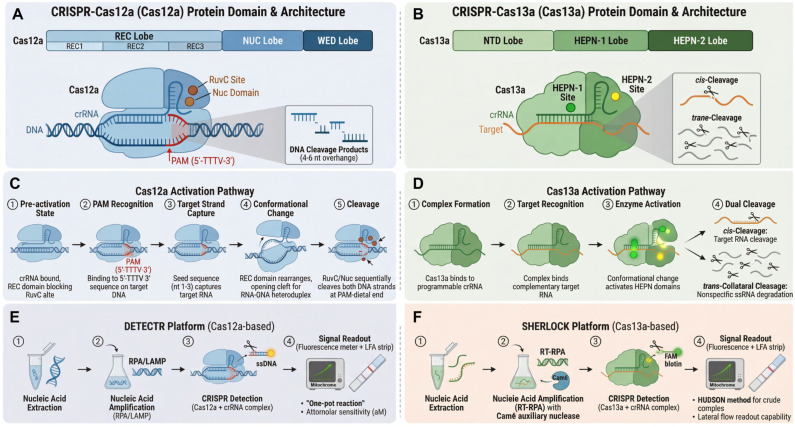
CRISPR-Cas12a and Cas13a: protein domain architecture, molecular mechanisms, and diagnostic platforms. (**A**,**B**) Schematic representation of the domain organization, target recognition, and collateral cleavage activities of Cas12a and Cas13a, highlighting their roles in nucleic acid detection. (**C**,**D**) Cas12a (**left**) is a DNA-targeting effector with a single RuvC domain; upon binding to target DNA via crRNA guidance and PAM recognition, it cleaves both the target and non-specifically degrades single-stranded DNA (ssDNA) reporters. Cas13a (**right**) is an RNA-targeting enzyme containing two HEPN RNase domains; its activation by target RNA leads to collateral cleavage of single-stranded RNA (ssRNA) reporters. (**E**,**F**) Both systems are coupled with isothermal amplification (e.g., RPA, LAMP) and enable visual readout (fluorescence or lateral flow strips) for rapid, sensitive, and field-deployable detection of swine viral pathogens.

The discovery of Cas12a (formerly Cpf1), a type V effector protein within the CRISPR/Cas system, represents a significant advance in genome-editing technology [27]. As a Class 2 CRISPR system component, Cas12a is an RNA-guided DNA endonuclease that differs from Cas9 in several key aspects. Cas12a requires only a single CRISPR RNA (crRNA) to form a functional complex, whereas Cas9 requires both a crRNA and a trans-activating crRNA (tracrRNA) [28]. Cas12a recognizes a 5′-TTTV-3′ protospacer adjacent motif (PAM) sequence, distinct from the 5′-NGG-3′ PAM required by Cas9 [29], thereby expanding the range of targetable genomic sites. In its cleavage mode, Cas12a generates cohesive ends with 4–5 nucleotide overhangs distal to the PAM site, in contrast to the blunt ends produced by Cas9 [30]. Structural studies reveal that Cas12a contains a single RuvC nuclease domain that accomplishes double-stranded DNA cleavage through one catalytic center, unlike Cas9, which requires two independent domains (HNH and RuvC) [30]. These characteristics have established Cas12a as a valuable addition to the genome-editing toolkit (Figure 2A).

At the molecular level, Cas12a exhibits crRNA processing properties distinct from those of Cas9 [31]. Cas12a can process its own precursor crRNA (pre-crRNA) via an endogenous ribonuclease active site in its WED domain, independent of tracrRNA or host factors such as RNase III. The mature crRNA for Cas12a (~42 nt) is shorter than the single-guide RNA (sgRNA, ~100 nt) or the crRNA–tracrRNA complex of Cas9, allowing more concise CRISPR array design and advantageous multiplex genome editing. Swarts et al. [32] elucidated the molecular mechanism of Cas12a through structural studies: after binding and processing crRNA, Cas12a adopts a pre-activation conformation where the crRNA seed sequence (nucleotides 1–5) is pre-ordered, while the REC domain blocks the RuvC catalytic site. Upon recognition of the 5′-TTTV-3′ PAM on target DNA, Cas12a facilitates target DNA alignment with the crRNA, inducing localized unwinding in the PAM-adjacent region [33]. Capture of the target DNA strand by the crRNA seed sequence triggers a conformational rearrangement, opening a central enzyme cleft to accommodate the RNA–DNA heteroduplex and exposing the RuvC active site. Conserved aromatic residues in the REC domain act as a “molecular ruler”, constraining the heteroduplex length to 20 base pairs and directing cleavage to the PAM-distal end of the R-loop. The conformational dynamics allow the single catalytic center to cleave the target and non-target strands sequentially, though the precise temporal relationship requires further elucidation. This model provides a theoretical foundation for understanding Cas12a’s gene-editing properties and its applications in nucleic acid detection (Figure 2C).

A defining feature of Cas12a is its robust trans-cleavage (collateral cleavage) activity upon target DNA recognition, enabling non-specific degradation of single-stranded DNA (ssDNA) [6]. This property has been harnessed for nucleic acid detection. When the Cas12a–crRNA complex binds its target DNA, the activated trans-cleavage activity cleaves short, fluorescent reporter ssDNA molecules labeled with a fluorophore–quencher pair, generating a detectable signal [33]. CRISPR-based detection typically involves four steps: (1) nucleic acid extraction, (2) nucleic acid amplification, (3) CRISPR detection, and (4) signal readout. Extraction can be performed rapidly using commercial kits. Amplification primarily employs isothermal techniques such as LAMP or RPA. The appropriate CRISPR system is then selected based on the target nucleic acid type. Finally, results are interpreted via fluorescence or other visual means. The DETECTR (DNA Endonuclease Targeted CRISPR Trans Reporter) technology, developed based on this principle, achieves attomolar (aM)-level sensitivity for viral genomic nucleic acids by combining Cas12a with isothermal amplification [6]. DETECTR offers several core advantages. First, its “one-pot” reaction system integrates isothermal amplification and CRISPR detection in a single tube [34,35], reducing aerosol contamination risks associated with qPCR. Second, results can be visually interpreted using portable LED devices, minimizing reliance on specialized equipment. Finally, the assay can be completed within one hour. These attributes underscore DETECTR’s value in outbreak response. Its high sensitivity, rapid turnaround, and field-deployability make it an ideal point-of-care testing (POCT) solution. By designing crRNAs targeting different viral genes, the Cas12a system can be developed into a multiplex detection platform. Studies show optimized systems can simultaneously identify 4–6 different pathogens in a single reaction [6,27,36,37], maintaining aM-level sensitivity with reaction times of 30–60 min, thereby enhancing detection throughput and efficiency (Figure 2E).

## 4. Principles and Technical Features of CRISPR-Cas13a-Based Detection

Beyond genome editing, the CRISPR-Cas system has emerged as a powerful platform for next-generation molecular diagnostics [38]. A pivotal breakthrough was the discovery of the collateral cleavage activity of the type VI effector Cas13a [7]. Unlike DNA-targeting Cas9, Cas13a is an RNA-guided ribonuclease whose activation triggers sequence-specific cleavage of target RNA (cis-cleavage) and nonspecific degradation of surrounding single-stranded RNA (ssRNA) molecules (trans- or collateral cleavage) [39,40]. The Cas13a protein comprises NUC lobes, a crRNA guide, and two HEPN (Higher Eukaryotes and Prokaryotes Nucleotide-binding) RNase domains responsible for RNA recognition and degradation. Mechanistically, LshCas13a cleaves ssRNA upon recognizing a target sequence (22–28 nucleotides) complementary to the spacer region of its crRNA [33] (Figure 2B). Activation requires guide-target RNA duplex formation. Target binding induces conformational changes in Cas13a, particularly rotations in the HEPN1 and Helical-2 domains, creating a central channel within the NUC lobe to accommodate a 28-base pair guide-target duplex resembling an A-type helix [41]. This rearrangement positions the HEPN RNase domains for cleavage (Figure 2D).

This inherent signal amplification mechanism—where a single target-binding event catalyzes the turnover of numerous reporter RNAs—forms the biochemical foundation for highly sensitive nucleic acid detection platforms [38]. The first diagnostic methodology to systematically harness this property was the Specific High-sensitivity Enzymatic Reporter unLOCKing (SHERLOCK) platform [9] (Figure 2F). Its standard workflow comprises three core stages: target amplification, Cas13a-mediated detection, and fluorescent signal generation. To achieve attomolar-level sensitivity, the initial nucleic acid target (DNA or RNA) is pre-amplified using an isothermal method, typically Recombinase Polymerase Amplification (RPA) or Reverse Transcription RPA (RT-RPA). For DNA targets, the RPA amplicon is transcribed into RNA via T7 RNA polymerase. The resulting RNA is incubated with a complex of Leptotrichia wadei Cas13a (LwaCas13a) and a programmable crRNA. Upon recognition of a complementary target sequence, activated Cas13a degrades a quenched fluorescent ssRNA reporter, separating the fluorophore from its quencher and yielding measurable fluorescence [38]. This system demonstrates single-nucleotide specificity and has been deployed to detect pathogens such as Zika and dengue viruses [9].

Subsequent engineering led to SHERLOCKv2, which introduced key innovations to enhance multiplexing, sensitivity, and point-of-care compatibility [33]. Major improvements included: (1) Multiplexed Detection: Using four orthogonal CRISPR effectors—LwaCas13a, *Prevotella* sp. MA2016 Cas13b (PsmCas13b), Capnocytophaga canimorsus Cc5 Cas13b (CcaCas13b), and *Acidaminococcus* sp. Cas12a (AsCas12a)—each with distinct dinucleotide cleavage preferences, enabled simultaneous detection of up to four targets. (2) Quantitative Capability: Using limited primer concentrations to prevent amplification saturation allowed semi-quantitative analysis. (3) Signal Amplification Cascade: Incorporating the auxiliary nuclease Csm6, activated by cyclic oligoadenylates produced during Cas13 collateral activity, added an extra amplification step, increasing sensitivity approximately 3.5-fold. (4) Lateral Flow Readout: The system was adapted for visual detection on lateral flow assay (LFA) strips. A reporter RNA conjugated with a fluorophore (e.g., FAM) and biotin is cleaved upon Cas13a activation; the released FAM-labeled fragment is captured by anti-FAM antibodies on gold nanoparticles, producing a visible test line, eliminating the need for fluorometric equipment [38].

To further simplify field applications, the Heating Unextracted Diagnostic Samples to Obliterate Nucleases (HUDSON) method was developed to enable direct detection from crude clinical samples (e.g., serum, saliva, urine). This protocol uses heat and chemical reduction to inactivate nucleases and release nucleic acids, bypassing conventional extraction [42].

The principal advantages of Cas13a-based biosensors include exceptional sensitivity (attomolar range), high specificity capable of distinguishing single-nucleotide variants, potential for quantitative and multiplexed analysis, and compatibility with instrument-free visual readouts—all favorable for point-of-care diagnostics. Limitations include dependence on a pre-amplification step, which adds time, cost, and complexity. While the requirement for a protospacer flanking site (PFS) can be mitigated by crRNA design, reliance on isothermal amplification remains a bottleneck. Additionally, achieving robust quantitative outputs and expanding multiplexing capacity beyond a limited number of targets require further development [38].

## 5. CRISPR-Cas12a/13a-Based Nucleic Acid Detection Methods for African Swine Fever Virus

African swine fever (ASF), caused by African swine fever virus (ASFV), is a highly contagious disease characterized by lethal hemorrhagic syndrome in domestic pigs and wild boars, with mortality rates approaching 100% [43]. Infected animals shed large amounts of virus through excreta and secretions, posing a persistent threat to the global swine industry [44,45]. First identified in Kenya in 1921, ASF spread to Portugal via contaminated food waste in 1957, subsequently affecting several Western European countries before being largely eradicated by 1995. The virus re-emerged in Georgia in 2007 after introduction from Africa, leading to persistent spread across Eastern Europe and into Nordic countries by 2014. Post-2018, the disease entered a phase of global dissemination, rapidly spreading to 11 countries in Asia and multiple African nations. China reported its first ASF outbreak in 2018, caused by a genotype II virulent strain highly homologous to Georgia-07 [46,47]. Surveillance in 2020 identified low-virulence genotype II variants lacking the EP402R gene, which can cause chronic, diagnostically challenging infections [48]. In 2021, a non-hemadsorbing genotype I low-virulence strain was isolated [49]. Most recently, genotype I/II recombinant viruses have been identified, showing 43.5% and 56.5% genomic homology to the respective parent genotypes. These recombinant strains are highly lethal and can breach immune protection conferred by existing genotype II vaccines, presenting a severe challenge to China’s ASF control framework [50]. The disease spreads via contaminated fomites and wild boar migration, and its global epidemiology has evolved through three major phases, highlighting the urgency for enhanced cross-border control and vaccine development. Notably, no fully safe and effective commercial vaccine has been approved for ASFV, and clinically validated therapeutic interventions are lacking. In this context, establishing rapid, accurate detection and surveillance systems has become a critical alternative strategy for ASF control.

**Figure 3:**
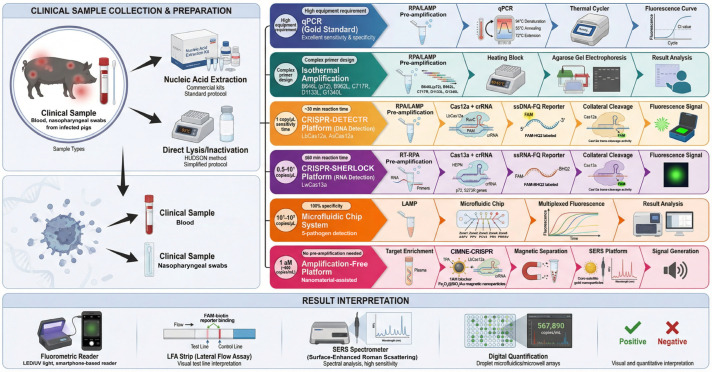
Technical workflow for ASFV nucleic acid detection. Samples collected from infected pigs (blood or nasopharyngeal swabs) are processed through inactivation and DNA extraction or lysis kit treatment. Subsequently, nucleic acid analysis can be performed using various techniques such as qPCR, isothermal amplification, CRISPR-based systems, biosensors, microfluidics, and nanomaterial-assisted amplification-free detection. Experimental results can be interpreted via fluorescence readout, lateral flow assay (LFA), surface-enhanced Raman spectroscopy (SERS), digital quantification.

Significant progress has been made in ASFV detection technologies, primarily encompassing traditional methods and emerging novel techniques (Figure 3). Traditional methods include immunological and molecular biological assays. Immunological methods, such as ELISA and lateral flow assays (LFA), offer simplicity and low cost but are limited by suboptimal sensitivity and accuracy for early infection detection [3,51]. Molecular methods, with quantitative PCR (qPCR) as the representative, are considered the gold standard for gene detection due to their excellent sensitivity and specificity. However, their reliance on specialized equipment and skilled personnel limits application in primary settings and resource-limited regions [4,52]. To overcome qPCR limitations, isothermal amplification techniques like LAMP [5,53,54] and RPA [55,56] have been developed. These techniques enable nucleic acid amplification without thermal cycling but have drawbacks: RPA suffers from relatively lower sensitivity, while LAMP faces challenges in primer design. Both methods are also susceptible to cross-contamination risks [5].

The introduction of CRISPR/Cas systems has brought revolutionary breakthroughs to ASFV detection. Studies have found that CRISPR-associated proteins such as Cas12a, Cas13a, and Cas14, upon specific target recognition, can activate non-specific trans-cleavage (collateral) activity. This property can be harnessed for specific identification of pathogen genetic sequences, facilitating rapid, highly sensitive, and low-cost early disease diagnosis [6,33,57,58]. CRISPR-based detection involves specific target recognition by a Cas enzyme-crRNA ribonucleoprotein complex, followed by signal amplification via trans-cleavage of fluorescent reporters [59]. This method does not require thermal cycling, making it suitable for POCT [60]. However, a significant technical bottleneck is the dependence on nucleic acid pre-amplification for sensitivity [61]. To overcome this, researchers have combined LAMP or RPA with CRISPR systems. The dual assurance of isothermal amplification and specific recognition enhances detection sensitivity and accuracy. For instance, CRISPR systems have been successfully applied to ASFV detection by integrating them with RPA isothermal amplification [62,63,64,65,66,67,68,69,70]. To address the cost and complexity of RPA, methods combining LAMP with CRISPR/Cas12a offer advantages. Compared to RPA-CRISPR systems, LAMP-CRISPR systems exhibit lower enzyme dependency, simpler operation (reducing manual steps by ~40%), and faster reaction times (within 30 min), achieving a detection sensitivity of 1 copy/μL [70]. However, dependence on pre-amplification remains a limitation for clinical practice [61].

To break through this barrier, researchers have developed digital detection methods. Integrating droplet microfluidics and microwell arrays with CRISPR systems has improved quantitative detection sensitivity for RNA and DNA [71,72]. For example, Ji et al. [73] combined LAMP with a CD-style microfluidic chip to achieve simultaneous detection of five pathogens: ASFV, porcine parvovirus (PPV), pseudorabies virus (PRV), PCV2, and PRRSV. The system’s limit of detection (LOD) for ASFV-MGF505-2R/P72, PPV, and PCV2 was 10^1^ copies/μL, and for ASFV-CD2v, PRV, and PRRSV, it was 10^2^ copies/μL, with 100% specificity and a coefficient of variation <5%. Zhu et al. [53] constructed a visual multiplex detection platform by integrating Hive-Chip technology with LAMP. This platform can simultaneously detect five ASFV genes (B646L, B962L, C717R, D1133L, and G1340L), with LODs of 30 and 50 copies/μL for synthetic DNA and simulated samples, respectively, and no cross-reactivity. However, microfluidic chips face challenges: complex fabrication processes require specialized skills and are time-consuming. Additionally, novel fluorescent reporter systems can enhance sensitivity but often involve expensive materials, increasing costs for routine CRISPR diagnostics.

To minimize false positives, recent research has focused on developing sensitive, simplified, amplification-free CRISPR/Cas detection systems [74]. Combining LbCas12a–crRNA ribonucleoprotein complexes with nanomaterials can enhance detection performance. For instance, electrostatic co-assembly with silicon nanoparticles increased stability in low-pollution environments threefold while improving fluorescence efficiency by three orders of magnitude [75]. Different Cas12a systems (e.g., LbCas12a and AsCas12a) exhibited differentiated trans-cleavage activities on gold nanoparticle surfaces. Moreover, LbCas12a trans-cleavage activity on nanoparticle surfaces depended on DNA strand density and length [76]. Integrating CRISPR/Cas12a systems with nanomaterial-based optical technologies like SERS provides a new approach for constructing efficient, amplification-free detection systems. For example, a core–satellite nanocluster structure centered on gold nanoparticles can greatly enhance SERS signals. Introducing magnetic response components improved detection sensitivity from 10 aM (~6000 copies/mL) to 1 aM (~600 copies/mL). This platform demonstrated excellent stability and repeatability, enabling precise target DNA detection in various biological environments [77]. Furthermore, Chen et al. [78] developed Fe_3_O_4_@SiO_2_/Au magnetic nanoparticles (MNPs), consisting of an Fe_3_O_4_ core, a surface-deposited layer of gold nanoparticles, and functionalization with 4-aminothiophenol, improving detection convenience. Building on CRISPR diagnostics and protein immobilization methods, Pal et al. [79] developed a covalently immobilized magnetic nanoparticles enhanced CRISPR (CIMNE-CRISPR) detection system, providing an innovative solution for accurate ASFV detection in resource-limited settings. Specifically, a crRNA was designed to target the B646L gene of ASFV (encoding the conserved viral protein vp72), containing the PAM required for recognition and cleavage by LbCas12a. The CIMNE-CRISPR system operates via: (1) target enrichment directly from samples without pre-amplification; (2) specific recognition and binding of target DNA by the RNP complex; (3) magnetic separation/purification to enrich the target complex and remove background; (4) signal transduction via addition of an ssDNA fluorescent-quencher probe, which is cleaved by activated LbCas12a, generating a fluorescent signal proportional to target concentration. Key technological breakthroughs of CIMNE-CRISPR include: innovative integration of CRISPR/Cas12a recognition, magnetic nanoparticle enrichment, and signal amplification; use of terephthalaldehyde (TPA) as a crosslinker, enhancing enzyme specificity and stability compared to traditional adsorption; and an innovative covalent immobilization strategy combined with 1-aminohexane (1AH) blocking to reduce non-specific interactions and contamination risks. Magnetic separation efficiency is markedly improved, and background noise is substantially reduced. This system demonstrates outstanding performance in complex matrices like porcine plasma: it achieves an LOD of 8.1 × 10^4^ copies/μL and a linear detection range spanning five orders of magnitude (10^5^–10^10^ copies/μL). Experimental data indicate excellent repeatability (RSD: 9.63%) and mutation tolerance, with accurate identification of ASFV DNA even when mutant sequences are present in 10,000-fold excess. The Fe_3_O_4_@SiO_2_ magnetic nanoparticle strategy enables cost-effective bioconjugation and expands applicability to various pathogens and complex matrices, making it valuable for on-site detection at primary veterinary stations and border quarantine.

The Cas13a system (specifically from Leptotrichia wadei, LwCas13a) has been harnessed for ASFV detection by coupling it with isothermal pre-amplification and lateral flow readouts. Upon recognition of target RNA complementary to its crRNA, activated LwCas13a non-specifically cleaves surrounding ssRNA, including a reporter molecule, enabling signal amplification. To detect ASFV DNA, reverse transcription recombinase polymerase amplification (RT-RPA) or recombinase-aided amplification (RAA) is first employed to amplify viral genomic targets (e.g., p72 or S273R genes), followed by in vitro transcription to generate RNA amplicons for Cas13a recognition [80,81,82].

Several optimized platforms have been reported. For example, one study integrated RPA, CRISPR-LwCas13a cleavage, and lateral flow strips (LFS) to achieve visual detection within 1 h, with a sensitivity of 10^1^ copies/µL for the p72 gene and no cross-reactivity with other common swine viruses [80]. Another approach used a “tube-in-tube” consumable design to combine RPA and Cas13a detection in a single closed tube, preventing aerosol contamination and achieving detection in 25 min with an LOD as low as 3 copies/µL for ASFV DNA [83]. Further innovations enabled high-throughput “sample-in-result-out” detection using lyophilized reagents and extraction-free sample processing, with an LOD of 0.5 copies/µL for ASFV [84].

Multiplexed detection strategies have also been developed by exploiting the orthogonal activities of Cas13a and Cas12a. One platform employed Cas13a for RNA reporter cleavage and Cas12a for DNA reporter cleavage in a single reaction, allowing simultaneous dual-gene detection of ASFV and SARS-CoV-2 with high specificity and sensitivity in clinical samples [82]. These systems often incorporate smartphone-based or portable fluorescence readers for point-of-care use.

In summary, CRISPR-Cas13a-based assays offer a rapid (≤60 min), sensitive (LOD 0.5–10^1^ copies/µL), specific, and instrument-light platform for ASFV detection. They are compatible with visual lateral flow readouts and can be integrated into contamination-free, one-pot, and high-throughput formats, making them promising tools for field surveillance and early outbreak control in resource-limited settings.

## 6. CRISPR-Cas12a/13a-Based Nucleic Acid Detection Methods for Other Swine Viral Pathogens

Beyond ASFV, CRISPR-Cas12a and Cas13a systems have been extensively adapted for diagnosing other economically significant swine viral pathogens. These platforms generally integrate isothermal pre-amplification—such as RPA, RAA, or RT-RPA—with the collateral cleavage activity of Cas12a (for DNA targets) or Cas13a (for RNA targets), followed by fluorescence or lateral-flow strip (LFS) readout.

### 6.1. Porcine Reproductive and Respiratory Syndrome Virus (PRRSV)

Both Cas13a- and Cas12a-based assays have been developed for PRRSV, an RNA virus. A Cas13a-coupled RPA method achieved visual detection at 37 °C with an LOD of 172 copies/µL and no cross-reactivity with other common swine viruses [85]. In parallel, a highly sensitive one-step RT-RPA-Cas12a assay was established, enabling single-copy detection of PRRSV RNA within 25 min in a single closed tube, with clinical validation showing full concordance with RT-qPCR [86]. This Cas12a assay utilized an optimized FAM-BHQ2 single-stranded DNA reporter and a conserved sgRNA targeting the Nsp2 gene, demonstrating superior sensitivity over conventional PCR and earlier Cas13a-based tests [86].

### 6.2. Foot-and-Mouth Disease Virus (FMDV)

For FMDV serotype O, an RT-RAA-Cas13a method was developed through systematic screening of primers and crRNAs. The optimized assay operates at 37 °C, exhibits an LOD of 19.1 copies/µL, and shows no cross-reactivity with Senecavirus A, PRRSV, PEDV, PCV2, CSFV, or pseudorabies virus (PRV). It performed reliably on simulated clinical samples (swab, tissue, serum) and can be read using either fluorescence or LFS [87].

### 6.3. Classical Swine Fever Virus (CSFV)

A Cas13a-based DIVA (differentiation of infected and vaccinated animals) platform was designed to discriminate between CSFV virulent strains and the attenuated vaccine strain HCLV. Combined with RT-RAA and HUDSON sample treatment, the method directly detected viral RNA without nucleic acid extraction, achieving LODs of 3.5 × 10^2^ copies/µL for the Shimen strain and 1.8 × 10^2^ copies/µL for HCLV. The assay showed 100% specificity against other swine viruses and high concordance with nested PCR in clinical spleen samples [88].

### 6.4. Porcine Rotavirus (PoRV)

For PoRV, an RAA-Cas12a assay was established to simultaneously detect G5 and G9 genotypes by targeting the conserved NSP3 gene. The assay achieved an LOD of 2.43 copies/µL within 30 min at 37 °C—tenfold more sensitive than qPCR—with no cross-reactivity to PoRV G3, G4, PEDV, PDCoV, or PRRSV. Clinical validation yielded a Cohen’s Kappa value of 0.952 compared to qPCR [89].

### 6.5. Porcine Parvovirus 7 (PPV7)

A rapid RPA-Cas12a visual assay was developed for PPV7, targeting the conserved NS1 region. After optimization of crRNA and ssDNA reporter concentrations, the assay attained an LOD of 100 copies/µL and specifically detected PPV7 without cross-reactivity. In testing 50 clinical lung tissue samples, it identified 29 positives (58%), outperforming conventional PCR (22 positives, 44%) [90].

### 6.6. Porcine Circovirus (PCV)

CRISPR-Cas12a/13a systems coupled with isothermal amplification (ERA, RPA, or LAMP) have been systematically applied for detecting PCV types 2, 3, and 4. These methods achieve single-copy/µL sensitivity, 100% specificity against other swine viruses (e.g., PRRSV, PEDV, PRV, CSFV), and complete concordance with qPCR in clinical samples. They are designed for equipment-free, visual, on-site use, substantially lowering the barrier for field deployment [91].

### 6.7. General Advantages, Technical Features and Remaining Challenges

Across these applications, combining isothermal amplification with CRISPR-Cas12a/13a consistently delivers high sensitivity (LODs from single-copy to ~10^2^ copies/µL), excellent specificity with no cross-reactivity to common swine pathogens, rapid detection within 25–60 min at constant temperature (37 °C), and full compatibility with point-of-care readouts such as portable fluorescence or lateral-flow strips. Moreover, these assays exhibit robust clinical performance, often achieving 100% concordance with gold-standard PCR/qPCR on field samples. Despite advantages, most current CRISPR-based assays still require nucleic-acid extraction, hindering true “sample-in, result-out” operation in resource-limited settings. Further integration of extraction-free protocols (e.g., HUDSON) and adoption of lyophilized reagent formats will be crucial to enhance practicality and deployability [86,88,91]. In summary, CRISPR-Cas12a and Cas13a have been successfully tailored for detecting a broad spectrum of swine viral pathogens—including PRRSV, FMDV, CSFV, PoRV, PPV7, and PCV—offering rapid, sensitive, specific, and field-deployable diagnostic solutions poised to transform disease surveillance and control in the global swine industry.

## 7. Discussion and Conclusions

CRISPR-Cas12a and Cas13a systems represent a transformative advance in detecting swine viral pathogens. By harnessing collateral cleavage activity and integrating isothermal amplification, these platforms achieve high sensitivity (LODs from single-copy to ~10^2^ copies/μL), excellent specificity, and rapid turnaround (typically 25–60 min) under constant temperature conditions [92]. Compatibility with visual readouts—such as fluorescence under portable UV/blue LED or lateral flow strips—enables deployment in resource-limited and field settings, addressing a critical gap between centralized laboratory testing and point-of-need diagnostics. It is important to note that while the CRISPR-Cas detection core itself is elegantly simple, the overall workflow still faces the universal challenge of efficient and field-robust nucleic acid extraction. Ongoing efforts to simplify this initial step are crucial for realizing the full point-of-care potential of this technology.

For ASFV, both Cas12a and Cas13a have been successfully applied in formats combining RPA/RAA with lateral flow or fluorescence detection, achieving sensitivities as low as 0.5 copies/μL and enabling high-throughput, contamination-free assays. Similarly, PRRSV detection has been accomplished with single-copy sensitivity using a one-step RT-RPA-Cas12a assay, outperforming earlier Cas13a-based methods. For CSFV, a Cas13a-based DIVA platform combined with HUDSON sample treatment allows discrimination between virulent and vaccine strains without nucleic acid extraction. Other viruses, including FMDV, PoRV, PPV7, and PCV, have also been detected with high accuracy and specificity using tailored CRISPR assays, demonstrating broad applicability across diverse swine pathogens.

### 7.1. Economic Considerations, Accessibility, and Challenges for Scale-Up

The main practical barrier to field deployment of CRISPR-Cas diagnostics remains nucleic acid extraction (e.g., column-based or magnetic bead kits), which is often labor-intensive and equipment-dependent, hindering fully integrated point-of-care workflows. Simplified lysis buffers and all-in-one devices offer promising alternatives [93]. Economically, the technology reduces capital expenditure by using heating blocks instead of thermocyclers, improving accessibility. Although Cas proteins and guide RNAs remain specialized, scaling production is lowering costs. Compared to qPCR—which benefits from standardization and economies of scale in central labs—CRISPR-based tests may be more cost-effective in low-to-medium throughput field settings, aided by lyophilized, stable reagents and minimal training requirements. Beyond per-test costs, the primary value lies in early pathogen detection, enabling rapid containment and avoiding large-scale losses from depopulation, trade restrictions, and production downtime. This cost–benefit proposition supports adoption across farm scales, enhances biosecurity, and promotes antimicrobial stewardship.

### 7.2. Application Positioning, Standardization Challenges, and Translational Pathways

#### 7.2.1. Intended Application Scenarios and Diagnostic Positioning

CRISPR-Cas12a/13a diagnostics are primarily positioned as a rapid field screening and triage tool, critical for outbreak investigation, pre-movement testing, and on-farm differential diagnosis. They also serve as a supplemental confirmatory method in resource-limited laboratories lacking qPCR access. Adoption as a primary official diagnostic for international reporting, however, requires extended validation and standardization under bodies such as World Organisation for Animal Health (WOAH). Commercialization currently follows a dual pathway. While designed for point-of-care use, the largest end-use segment remains hospitals/clinics (42.31% of 2024 revenue), supported by assay kits/reagents (61.93% share) and Cas12-based technology (47.03%), mainly in infectious disease diagnostics (61.60%). The market, valued at USD 502.64 million in 2024, is projected to grow at a CAGR of 15.41% through 2033 (USD 1786.56 million). The fastest-growing segment is homecare/at-home testing, with Asia Pacific as the fastest-growing region, signaling a clear shift toward decentralized, field-ready applications [94].

#### 7.2.2. Current Landscape: Standardization Hurdles and Commercialization Progress

Routine adoption faces significant standardization challenges, notably the absence of universally accepted reference protocols, leading to inter-laboratory variability. Multicenter clinical trials and head-to-head comparisons with established methods are essential to establish reproducibility. A key obstacle to integrated CRISPR-Cas diagnostics remains sample preparation (lysis and extraction), with nearly 90% of reviewed systems leaving this step off-device [95]. Commercialization is further hindered by ambiguous regulatory classification (laboratory-developed tests (LDTs) vs. in vitro diagnostics (IVDs)), which delays approval, and by costly validation requiring tens of thousands of clinical samples for certifications such as FDA/CE. Manufacturing consistency is also challenged by batch-to-batch variability in Cas protein activity exceeding 20%, affecting reliability. Although regulatory pathways specific to CRISPR are emerging and innovations like lyophilized reagents are being developed, these combined barriers substantially slow the translation of prototypes into standardized commercial products. Commercial kit availability is advancing in human health (market ~USD 3.1 billion in 2024), with companies like Mammoth and Sherlock having obtained EUAs for SARS-CoV-2 tests. For veterinary applications, however, fully validated IVD kits for swine diseases remain scarce, with most products still at the RUO or early-access stage [95].

#### 7.2.3. Multifaceted Challenges on the Translational Pathway

Beyond the previously discussed bottleneck of nucleic acid extraction, several layers of challenges must be addressed. The path to routine field deployment is hindered by a cascade of interconnected challenges. Beyond the foundational need for simplified sample preparation, achieving reliable multiplexing without cross-talk and ensuring robustness against complex sample inhibitors commonly found in clinical matrices remain significant technical hurdles. Furthermore, clinical validation is challenged by target heterogeneity and the lack of universally accepted reference standards, necessitating extensive multicenter trials. Finally, user adoption depends on overcoming operational complexity—streamlining the workflow into a rapid, sub-30 min “sample-to-result” process is critical for point-of-care utility. Addressing these analytical, validation, and usability barriers in parallel is essential for translation [93].

#### 7.2.4. Adoption Pathways and Impact on Farm Management

CRISPR-Cas diagnostics will be adopted along distinct pathways shaped by existing infrastructure. In well-equipped reference laboratories, they may serve as a rapid frontline triage tool, while qPCR remains for confirmation. For local labs with limited resources, the technology offers a transformative alternative, providing molecular-level accuracy with minimal equipment (e.g., heat block and reader), thereby democratizing access to rapid diagnostics. On-farm deployment could revolutionize swine health management by enabling pathogen-specific decision-making within hours. This supports targeted interventions and facilitates ultra-early “precise depopulation” (or “selective culling”) strategies to contain outbreaks before widespread dissemination, substantially reducing potential losses. Furthermore, frequent and cost-effective testing enables robust monitoring, traceability, and data-driven biosecurity programs [95]. Several technical challenges persist, including the need for integrated nucleic acid extraction and limited multiplexing capacity in complex samples. Innovations such as extraction-free protocols, lyophilized reagents, and amplification-free CRISPR platforms are promising yet require further optimization for field-ready use.

In conclusion, CRISPR-Cas12a/13a systems represent a paradigm-shifting technology with the potential to democratize rapid and accurate diagnosis. This review not only elucidates the mechanistic principles and recent advancements but also critically evaluates its practical application scenarios, translational hurdles, and future pathways toward integration into modern swine disease management systems. Their high performance, speed, and visual readout capability position them as ideal candidates for point-of-care surveillance, outbreak response, and routine monitoring in diverse agricultural settings. Ultimately, the adaptation of this technology must be contextualized within diverse global pig production systems. In highly intensive, technology-driven systems (e.g., North America, Europe), it will integrate as a sentinel and precision tool within existing sophisticated biosecurity networks. In rapidly intensifying, large-scale systems (e.g., parts of Asia, South America), it holds transformative potential to leapfrog traditional diagnostic infrastructure gaps. For smallholder or resource-constrained settings, its value may be realized through innovative shared-service models (e.g., mobile diagnostic units), bringing advanced diagnostics within reach [95]. Realizing this full potential requires a concerted, interdisciplinary effort to tackle the practical challenges of standardization, commercialization, and field validation outlined herein, thereby bridging the gap between innovative molecular science and actionable veterinary solutions. Ongoing innovation in sample preparation, reagent stabilization, and multiplex detection will further solidify the role of CRISPR-based systems in safeguarding swine health and supporting the sustainability of the global pork industry.

## Data Availability

No new data were created or analyzed in this study. Data sharing is not applicable to this article.
